# Divergent microbial strategies under saline–alkali stress: a comparative study of bacterial and fungal communities in four agro-pastoral habitats

**DOI:** 10.3389/fmicb.2026.1809796

**Published:** 2026-05-07

**Authors:** Zhijie Tian, Xueying Jia, Xinyue Yang, Jing Lin, Hongchao Sun, Xinlu Zhang, Yang Yang

**Affiliations:** 1 Department of Life Sciences, Xinzhou Normal University, Xinzhou, China; 2 School of Geographical Sciences, Taiyuan Normal University, Jinzhong, China; 3 School of Life Science, Shanxi University, Taiyuan, China; 4 College of Food Science and Engineering, Shanxi Agricultural University, Jinzhong, China

**Keywords:** agro-pastoral ecotone, co-occurrence network, functional prediction, halophilic microorganisms, microbial community, PLS-PM, saline–alkali stress

## Abstract

**Introduction:**

Microorganisms regulate nutrient cycling and ecosystem stability in saline–alkali ecosystems. However, their adaptation mechanisms and metabolic functions to saline–alkali stress in agro-pastoral ecotones remain unclear.

**Methods:**

We characterized bacterial and fungal communities in four typical saline–alkali habitats in the agro-pastoral ecotone of the Loess Plateau [saline–alkali flat (SAF), natural grassland, maize cropland, and shrubland] via Illumina MiSeq sequencing. We analyzed community compositions, α-diversity, co-occurrence network characteristics, and functional group differences, using Mantel tests, redundancy analysis (RDA), and partial least squares path to examine environmental drivers of microbial structure and function.

**Results:**

Saline–alkali stress significantly altered soil physicochemical properties (*P* < 0.05), with SAF exhibiting higher pH, EC, Na^+^, and low available nutrients. Bacteria α-diversity differed significantly among habitats (*P* < 0.05), suggestive of sensitivity to saline–alkali stress. Under such stress, bacterial co-occurrence networks simplified with intensified interspecific competition, whereas fungal networks maintained high modularity (0.77–0.81) and were dominated by positive correlations (>93%), reflecting a synergistic coexistence. Functionally, saline–alkali stress inhibited bacterial chemoheterotrophy, aerobic ammonia oxidation, and nitrification. Saline–alkali stress also promoted fermentation, phototrophy, and nitrate reduction and jointly drove microbial functional expression, with pH, available phosphorus, and soil organic carbon (SOC) identified as key drivers (*P* < 0.05).

**Conclusion:**

We identified adaptive strategies and functional differentiation of bacterial and fungal communities under saline–alkali stress. These findings provide important insights for the sustainable management and ecological restoration of saline–alkali lands in agro-pastoral ecotones.

## 1 Introduction

Soil salinization is a global form of land degradation that poses a serious threat to agricultural productivity, soil function, and ecosystem stability. The global extent of salinized land exceeds 1.381 billion hectares (Food Agriculture Organization of the United Nations, 2024), of which the total area of saline–alkali soil in China amounts to 99.13 million hectares (Nadeem and Liu, 2023). In agro-pastoral ecotones, soil salinization has intensified under the combined pressures of climate change and anthropogenic activities, and is limiting the sustainable development of regional agriculture and animal husbandry. Unlike coastal saline lands shaped by marine salt intrusion and inland arid saline areas dominated by permanent evaporative salt accumulation (Zhang et al., 2026), these ecotones are characterized by pronounced seasonal water stress and diverse land-use conversions, resulting in marked spatiotemporal heterogeneity in salt accumulation (Liu H. et al., 2025). The high salinity and alkalinity of saline–sodic soils cause soil particle dispersion and expansion, structural degradation, reduced fertility, and impaired microbial functioning (Zahedifar, 2020). As core components of ecosystems, soil microorganisms participate in key ecological processes including organic matter decomposition, nutrient cycling, and soil structure formation (Fernandes et al., 2022; Philippot et al., 2023). Their community structure and functional responses to saline–alkali stress directly influence ecosystem stability and restoration potential. Under prolonged saline–alkali stress, microbial communities undergo adaptive evolution, including increase proportions of salt-tolerant genotypes and phenotypic groups (Rath et al., 2019). These adaptations primarily occur through differential impacts on bacterial and fungal communities via adjusted species–environment matching relationships (Gao et al., 2022). Therefore, systematic analysis of microbial community responses and adaptation mechanisms to saline environments is important for understanding and enhancing ecosystem functions and service capacities in saline lands. Therefore, systematically analyzing microbial community responses and adaptation mechanisms to saline environments holds significant importance for scientifically understanding and enhancing ecosystem functions and service capacities in saline lands.

Elevated salinity inhibits multiple core soil ecosystem processes, including carbon and nitrogen degradation, nitrogen fixation, anaerobic ammonium oxidation, phosphorus uptake and transport, and sulfur metabolism (Zhang et al., 2021; Yang et al., 2023). Metagenomic sequencing has consistently demonstrated that elevated salinity significantly reduces the abundance of key nitrogen cycling genes, such as those involved in ammonia oxidation (*amo*) and nitrite oxidation (*nxr*), while reducing the abundance of phosphorus transformation-related genes (*pho*D and *gcd*) through altered soil properties, thereby weakening the bioavailable nutrient supply capacity (Huang et al., 2022; Zhang et al., 2025). However, these inhibitory effects are not universal across microbial functions. Some reports have revealed positive responses of key functional genes to soil salinization stress, including carbon sequestration, denitrification, and organophosphate degradation (Zhang et al., 2021; Fan et al., 2023). This trend was confirmed in the Qinghai–Tibet Plateau, where high-salinity environments promoted microbial functions related to carbon degradation, denitrification, and sulfur oxidation (Li et al., 2021).

Decreased complexity and stability of microbial ecological networks can impair the capacity of ecosystems to provide essential functions and services (Wagg et al., 2019; Yuan et al., 2021). Investigations in agro-pastoral ecotones have revealed that saline–alkali stress tends to simplify bacterial networks while increasing the complexity of fungal networks, thereby contributing to enhanced overall network stability and optimized soil biogeochemical cycling (Wang et al., 2025). Halophytes play crucial regulatory roles in microbial community assembly and functional enhancement. Halophytes such as *Salicornia europaea* and *Suaeda glauca* can significantly increase soil carbon and nitrogen cycling when compared with that in bare saline lands. Their rhizosphere soils are enriched with *Pseudomonadota*, which enhance plant salt tolerance while concentrating functional microbial groups such as phototrophic bacteria and sulfate-reducing bacteria (Liang et al., 2025). Multiple lines of evidence have confirmed that increased plant diversity promotes the recruitment of diverse soil microbes and enhances pathogen antagonism by increasing the abundance of beneficial bacteria (Mommer et al., 2018; Wang et al., 2023). Survey data from saline soils with different vegetation have supported this conclusion; agricultural and forest saline soils were dominated by *Actinomycetota* and *Pseudomonadota*, whereas grassland saline soils showed significantly higher abundances of *Actinomycetota, Bacteroidota*, and *Gemmatimonadota* (Peng et al., 2017). However, recent research has predominantly focused on single microbial groups or extreme environments, leaving the synergistic response mechanisms and functional differentiation patterns of bacterial and fungal communities in the heterogeneous habitats of agro-pastoral ecotones unclear.

Focusing on the agro-pastoral ecotone of the Loess Plateau, we selected four typical saline–alkali habitats, including natural grassland, maize farmland, saline–alkali flat (SAF), and shrubland. We integrated high-throughput sequencing, co-occurrence network analysis, and functional prediction to systematically investigate how saline–alkali stress shapes microbial community assembly, interaction patterns, and ecosystem functioning in agro-pastoral ecotones. We hypothesized that: (1) bacterial and fungal communities exhibit differential sensitivity to saline–alkali stress; (2) microbial networks display distinct adaptive strategies under stress; and (3) microbial functional differentiation is co-regulated by saline–alkaline stress and soil nutrients. These findings are expected to deepen understanding of microbial ecological adaptation mechanisms in agro-pastoral saline–alkali soils, providing a scientific basis for enhancing ecosystem functions and sustainable management of saline–alkali lands in the region.

## 2 Materials and methods

### 2.1 Study site and soil sampling

The study area is in Shanyin County, Shuozhou City, Shanxi Province, China (39°11′-39°47′ N, 112°25′-113°04′ E), situated within the agro-pastoral ecotone of the northern Loess Plateau. The region is characterized by a temperate continental monsoon climate, with a mean annual temperature of 7.0 °C, annual precipitation of 350–450 mm (concentrated in the summer) and high annual evaporation reaching 1,800 mm. Due to seasonal groundwater fluctuations, salts tend to accumulate in the topsoil of this area, resulting in a compact soil structure, poor permeability, and salinization characteristics dominated by carbonates and bicarbonates. The soil pH in this area ranges widely from 8.0 to 11.0, with large variations in electrical conductivity (EC), making it a typical representative of the inland arid saline–alkali region in Northern China.

### 2.2 Soil physicochemical properties

**Figure 1:**
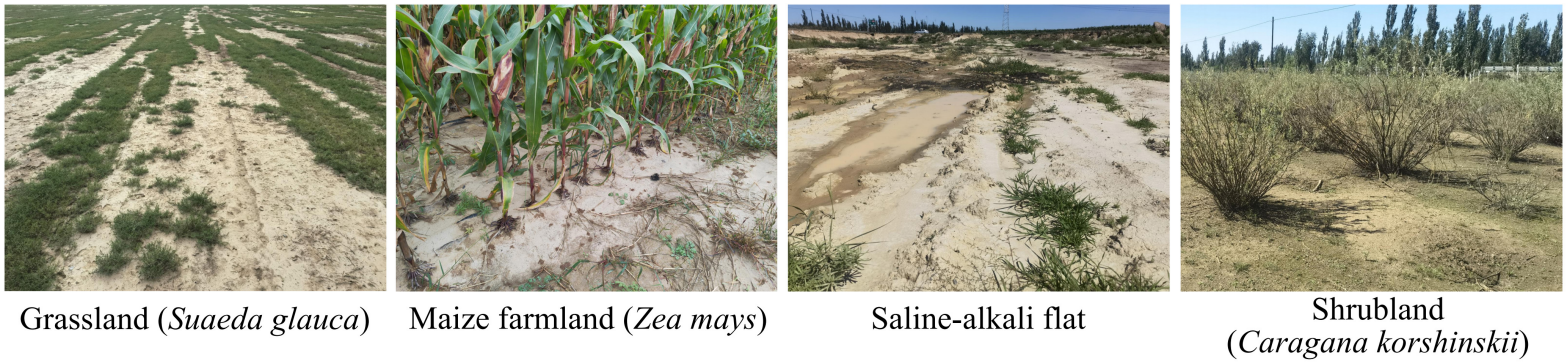
Vegetation characteristics of the saline–alkali sampling site.

We studied four typical saline–alkaline habitats along an ecological gradient of salinization and vegetation degradation: (1) SAF, a severely degraded, bare land with approximately 5% vegetation coverage, characterized by exposed soil surfaces often accompanied by white salt crusts and minimal anthropogenic disturbances; (2) grassland (Grass), a moderately degraded saline–alkaline area dominated by *Suaeda glauca*, with vegetation coverage of approximately 40%−50%; (3) maize farmland (Maize), moderately degraded saline–alkaline land cultivated with maize (*Zea mays*), with approximately 60%−70% vegetation coverage during the growing season, subjected to conventional nitrogen, phosphorus, potassium fertilization and irrigation management, representing agriculturally improved saline–alkaline land; and (4) shrubland (Shrub), lightly degraded saline–alkaline area dominated by the planted species *Caragana korshinskii*, with approximately 30%−40% vegetation coverage. These saline–alkaline sites exhibited distinct differences in salinization degree and ecological status, collectively representing a degradation gradient from severely degraded, bare land to lightly vegetated areas (Figure 1).

Soil sampling was conducted in September 2024. At the time of sampling, there had been no rainfall for at least 1 week. Five replicate plots were established. Within each plot, five subsamples (0–20 cm) were collected along an “S” shaped pattern, pooled into a single composite sample after removing litter and roots, and split for analysis. Fresh soil was stored at −80 °C for DNA extraction, whereas air-dried soil was sieved (< 2 mm) for physicochemical analysis. The soil pH and EC were measured in a 1:5 (w/v) soil–water suspension. We determined soil organic carbon (SOC) via dichromate oxidation, total nitrogen (TN) via Kjeldahl digestion, and available nitrogen (AN) via alkaline hydrolysis. Total phosphorus (TP) was measured by performing HClO_4_-H_2_SO_4_ digestion with subsequent Mo–Sb colorimetry, and available phosphorus (AP) was determined via NaHCO_3_ extraction followed by Mo–Sb colorimetry. Water-soluble cations (Na^+^, Ca^2+^, Mg^2+^ and K^+^) were measured using an inductively coupled plasma instrument (ICP-PQ9000, Analytik Jena, Germany), and the sodium adsorption ratio (SAR) was calculated as follows:


$$\begin{array}{l} SAR\;=\;\frac{Na^{+}}{\sqrt{\left(Ca^{2+}+Mg^{2+}\right)/2}} \end{array}$$ (1)

where the concentrations of Na^+^, Ca^2+^, and Mg^2+^ are all expressed in mmol/L.

### 2.3 Soil microbial community amplicon sequencing

The soil microbial community structure was characterized by performing high-throughput amplicon sequencing. Total genomic DNA was extracted from 0.25 g of fresh soil using a PowerSoil DNA Isolation Kit. For each bacterial community studied, the V3–V4 hypervariable region of the 16S rRNA gene was amplified by polymerase chain reaction (PCR) using the universal primers 338F (5′-ACTCCTACGGGAGGCAGCAG-3′) and 806R (5′-GGACTACHVGGGTWTCTAAT-3′). The thermocycling conditions comprised an initial denaturation at 95 °C for 3 min; 35 cycles of denaturation at 95 °C for 30 s, annealing at 55 °C for 30 s, extension at 72 °C for 30 s; and a final extension at 72 °C for 5 min. For the fungal community, the internal transcribed spacer 1 region was amplified using the primers ITS1F (5′-CTTGGTCATTTAGAGGAAGTAA-3′) and ITS2R (5′-GCTGCGTTCTTCATCGATGC-3′). The same PCR program was used, except that annealing occurred at 53 °C. After purification and quantification, the amplified products were subjected to 2 × 300 base pair (bp), paired-end sequencing on an Illumina MiSeq platform (Illumina, USA). Raw sequences were processed using QIIME2 (version 2020.11). Quality filtering removed bases with a quality score of *q* < 20, truncated reads with lengths below 200 bp, and excluded sequences containing ambiguous bases (Ns). Chimeric sequences were identified and removed using UCHIME, including *de novo* chimera detection and reference-based detection against the SILVA and UNITE databases (related to bacteria and fungi, respectively). The processed sequences were clustered at 97% sequence similarity using the VSEARCH algorithm to generate operational taxonomic units (OTUs) for both bacteria and fungi. After quality filtering and rarefaction, the mean number of bacterial reads per sample was 67,311 ± 3,280 (mean ± standard deviation [SD]), and the mean number of fungal reads per sample was 76,920 ± 4,871 (mean ± SD). Before diversity analysis, all samples were rarefied to an even depth of 30,000 sequences per sample to eliminate the influence of uneven sequencing depth on the results. Taxonomic assignment was performed against the SILVA 138 (bacteria) and UNITE 8.0 (fungi) databases from the phylum to the species level, and the relative abundance of each taxon was calculated at each taxonomic rank.

### 2.4 Statistical analysis

All statistical analyses were performed in R software (version 4.5.0), unless otherwise specified. Differences in soil properties and microbial α-diversity among sites were assessed using one-way analysis of variance (ANOVA) followed by Tukey's honestly significant difference test (*P* < 0.05). Prior to ANOVA, normality was assessed using the Shapiro–Wilk test, and variance homogeneity was tested using Levene's test. For data that did not meet the assumptions of normality or homogeneity of variances, the Kruskal–Wallis non-parametric test was applied, or data transformation was performed. Venn diagrams were generated with the “ggvenn” package (v0.1.19) to illustrate shared and unique bacterial OTUs and fungal ASVs across soil types. The online platform (www.cloudtutu.com.cn) was used to plot dominant phylum/genus abundances, analyze α-diversity indices (Shannon, Simpson, and Pielou), and perform Mantel tests for microbial community-environment relationships. Before analyzing and visualizing α-diversity indices, all samples were subjected to rarefaction to ensure comparability among samples, with rarefaction depths set at 38,000 reads per sample for bacteria and 35,000 reads per sample for fungi.

The “psych” package (v2.5.6) was used to screen phylum-level taxa with relative abundance >0.1%. Microbial co-occurrence networks were constructed by calculating Spearman's rank correlation coefficients, with |*r*| > 0.5 and *P* < 0.05 as the threshold. This threshold (|*r*| > 0.5) corresponds to moderate-to-strong correlations, which effectively filters out weak correlations caused by random noise or extremely low-abundance species while retaining sufficient biological information, thereby reducing the proportion of false-positive edges in the network. Networks were visualized in Gephi v0.10.1 to calculate topological parameters (nodes, edges, degree, density, modularity, clustering coefficient, path length and positive/negative correlations). To verify the non-randomness of the co-occurrence network structures, we performed null model analysis using a configuration model that preserved the degree sequence. Based on the edge lists for each saline–alkali soil type, undirected networks were constructed using the “igraph” package. To assess network non-randomness, 999 random networks were generated while preserving node degree distributions. Topological indices were calculated for each random network to construct a null distribution. The observed networks metrics were then compared with the corresponding null model means, and the probability of the observed values exceeding random expectations was calculated using a one-tailed test. Networks were considered to exhibit deterministic (non-random) structure when *P* was < 0.05.

To identify key environmental drivers of microbial functional groups, variance inflation factors (VIFs < 10) were calculated using the “vegan” (v2.7.1) and “car” (v3.1.3) packages to remove collinear variables, followed by redundancy analysis (RDA) in Canoco 5. Microbial functions were predicted using the “microeco” (v1.16.0) and “tidyverse” (v2.0.0) packages. Bacterial functions were annotated using the FAPROTAX Database, whereas fungal trophic modes were classified using FUNGuild. Dominant functional groups were characterized by a relative abundance of > 0.5% for both bacteria and fungi. To explore the pathways through which soil saline–alkali stress and nutrients affect soil microbial communities and functions, partial least squares path modeling (PLS-PM) was performed using the “plspm” package (v0.5.2). The model incorporated two exogenous latent variables (saline–alkali stress and soil nutrients), two endogenous latent variables (bacterial and fungal communities), and two outcome variables (bacteria and fungi functions). Saline–alkali stress was represented by pH, EC, Na^+^ and SAR, whereas soil nutrients were represented by SOC, TN, AN, TP, and AP. Microbial community structure was characterized by the relative abundances of dominant phyla (relative abundance > 1%) and their diversity indices. To improve the stability of the microbial parameters, principal component analysis was performed separately on microbial abundance and functional datasets, and the resulting principal component scores were used as composite indicators in the PLS-PM. Model performance was assessed using the goodness-of-fit index, with values exceeding 0.5 and 0.7 indicating good or excellent fit, respectively.

## 3 Results

### 3.1 Soil physicochemical characteristics

Significant differences existed in the physicochemical properties among the four types of saline–alkali soils (Table 1). SAF soils exhibited relatively high EC (1,971.20 ± 113.79 μS/cm), pH (10.34 ± 0.05), Na^+^ content (3.45 ± 0.17 g/kg) and SAR (17.88 ± 1.18) values, along with the highest SOC (5.70 ± 0.76 g/kg). However, the TN, TP, AN and AP contents were all comparatively low, indicating a pattern of high salinity, high alkalinity, high Na^+^ content, high organic carbon but limited available nutrients. Grass and Maize soils showed relatively high pH values (10.43 and 9.94, respectively), with the highest AN (81.89 mg/kg) found in Grass and the highest AP (7.48 mg/kg) in Maize. Shrub soils had the lowest Na^+^ content (0.06 ± 0.05 g/kg) and minimal cation levels overall, as well as the lowest EC (113.28 ± 9.65 μS/cm) and SAR (0.28 ± 0.23c) value, indicating the mildest degree of salinization and relatively balanced nutrient availability.

**Table 1:** Physical and chemical properties of soils under saline–alkali stress.

Soil type	SOC	TP	AP	TN	AN	pH
	g/kg	mg/kg	mg/kg	g/kg	mg/kg	
Grass	2.14 ± 0.14b	396.69 ± 10.05a	3.73 ± 0.27b	1.01 ± 0.04a	81.89 ± 6.62a	10.43 ± 0.03a
Maize	2.46 ± 0.04b	395.74 ± 6.63a	7.48 ± 0.46a	1.08 ± 0.03a	42.59 ± 2.36b	9.94 ± 0.15b
SAF	5.70 ± 0.76a	332.26 ± 3.51b	1.47 ± 0.13c	0.70 ± 0.02b	42.96 ± 6.29b	10.34 ± 0.05a
Shrub	3.16 ± 0.14b	349.24 ± 4.34b	1.47 ± 0.31c	0.69 ± 0.04b	74.27 ± 7.77a	8.59 ± 0.09c
**Soil type**	**EC**	**Na** ^+^	**Ca** ^2+^	**Mg** ^2+^	**K** ^+^	**SAR**
	μ**S/cm**	**g/kg**	**g/kg**	**g/kg**	**g/kg**	
Grass	799.40 ± 40.12b	0.80 ± 0.09b	0.023 ± 0.002d	0.005 ± 0.001b	0.007 ± 0.001b	6.88 ± 0.86b
Maize	514.00 ± 9.56c	1.06 ± 0.04b	0.032 ± 0.003c	0.005 ± 0.002b	0.013 ± 0.003b	7.83 ± 0.42b
SAF	1,971.20 ± 113.79a	3.45 ± 0.17a	0.060 ± 0.003b	0.016 ± 0.002a	0.042 ± 0.004a	17.88 ± 1.18a
Shrub	113.28 ± 9.65d	0.06 ± 0.05c	0.077 ± 0.001a	0.012 ± 0.001a	0.002 ± 0.000b	0.28 ± 0.05c

Different letters (a, b, c) indicate significant effects. SOC, soil organic carbon; TP, total phosphorus; AP, available phosphorus; TN, total nitrogen; AN, available nitrogen; EC, electrical conductivity; SAR, sodium adsorption ratio. SAF means the saline–alkali flat.

### 3.2 Soil microbial community composition

**Figure 2:**
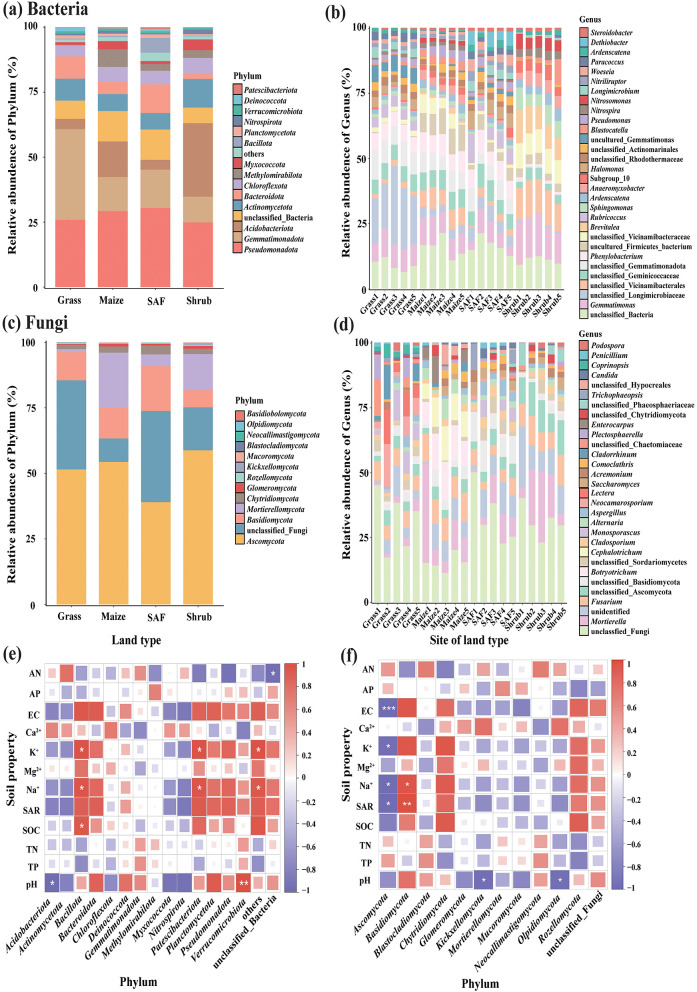
Bacterial and fungal community composition in saline–alkali soils. **(a)** and **(c)** phylum levels averaged across sites for each land type. **(b)** and **(d)** genus levels across different sites. **(e)** and **(f)** correlation analysis between soil physicochemical properties and bacterial and fungal, respectively. Red and blue colors in panels **(e)** and **(f)** indicate positive and negative correlations, respectively (*P* < 0.05).

High-throughput sequencing revealed 39,118 bacterial OTUs, covering 34 phyla, 100 classes, 316 orders, 690 families, 1,355 genera and 1,700 species. The dominant bacterial phyla (relative abundance > 5%) included *Pseudomonadota, Gemmatimonadota, Actinomycetota, Bacteroidota, Acidobacteriota* and *Chloroflexota* (Figure 2a). *Pseudomonadota* was the dominant phylum across all soil types, with an average relative abundance of 27.63% and higher proportions in SAF (30.48%) and Maize (29.28%) soils. *Gemmatimonadota* was the second most dominant phylum, with an average relative abundance of 18.15% and a notably higher abundance in Grass soils (34.90%). *Bacteroidota* exhibited the highest abundance in SAF soil (11.04%), whereas *Actinomycetota* and *Acidobacteriota* showed enrichment in Shrub soil. *Bacillota* (*r* = 0.96, *P* < 0.05) and *Patescibacteriota* (*r* = 0.96, *P* < 0.05) correlated significantly positively with soil Na^+^ content, as did *Verrucomicrobiota* with soil pH (*r* = 0.99, *P* < 0.01) (Figure 2e). At the genus level, *Gemmatimonas* and *Phenylobacterium* were the dominant groups in saline–alkali soils. Additionally, *Brevitalea* (10.03%) and *Sphingomonas* (6.30%) were relatively enriched in Shrub soils, whereas *Rubricoccus* (6.31%), *Halomonas* (4.76%) and *Dethiobacter* (4.18%) showed higher relative abundances in SAF soils (Figure 2b).

A total of 5,279 fungal ASVs were annotated, spanning 18 phyla, 52 classes, 103 orders, 232 families, 467 genera and 658 species. The dominant phyla (relative abundance >1%) were *Ascomycota, Basidiomycota, Mortierellomycota* and *Chytridiomycota*. *Ascomycota* was the overwhelmingly dominant group and the most abundant in Shrub soils (58.77%) and least in SAF soils (39.00%; Figure 2c). *Basidiomycota*, the second most dominant phylum, was most abundant in SAF soils (17.08%) and least abundant in Shrub soils (6.52%). The abundance of *Basidiomycota* correlated significantly positively with soil Na^+^ (*r* = 0.97, *P* < 0.05) and SAR (*r* = 0.99, *P* < 0.01; Figure 2f). At the genus level, *Mortierella* and *Fusarium* predominated across all saline–alkali soils. Grass soils featured *Neocamarosporium* and *Lectera*, whereas Maize soil was enriched for *Botryotrichum* and *Cephalotrichum* (Figure 2d).

### 3.3 Soil microbial community diversity

**Figure 3:**
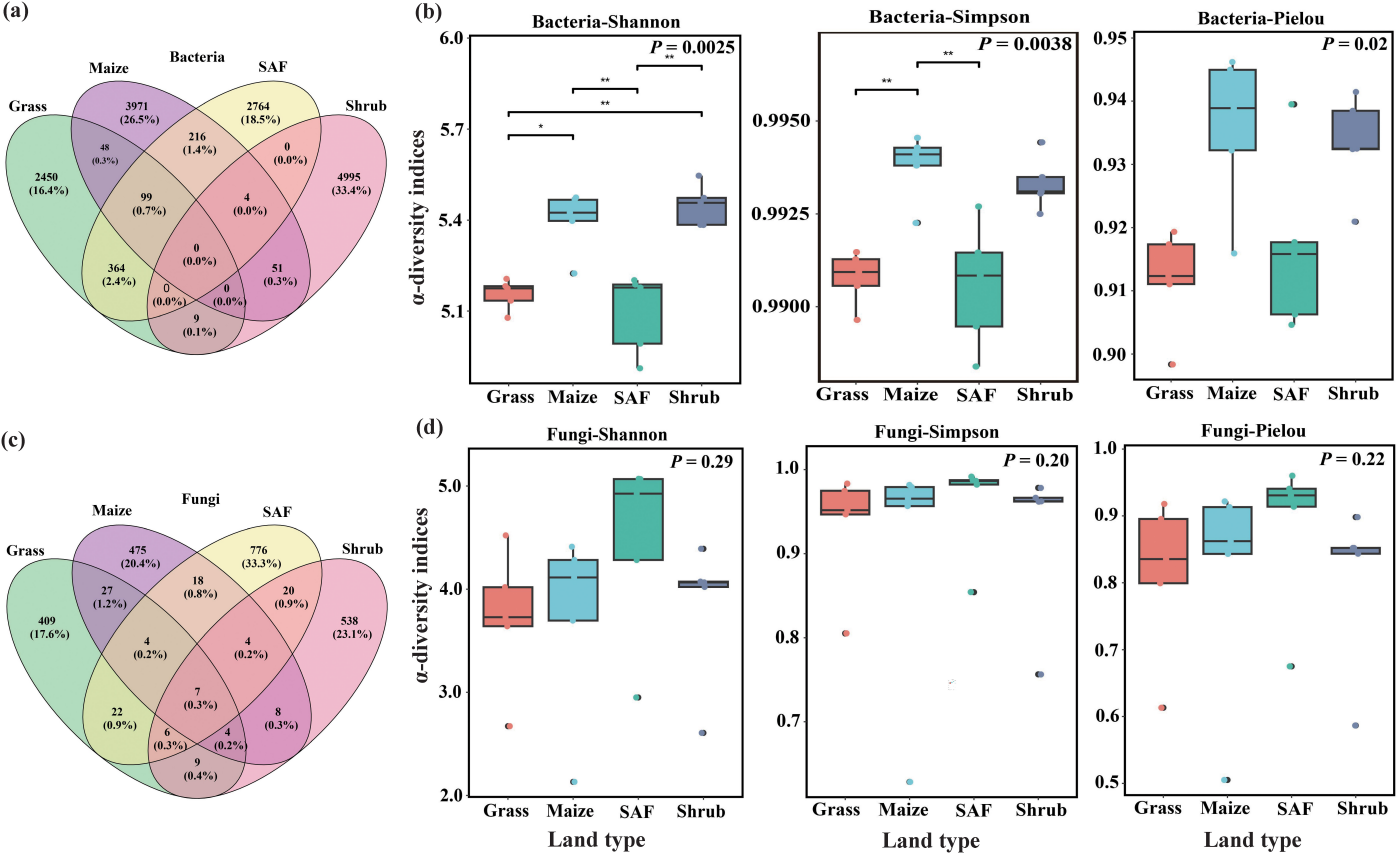
Venn diagram and alpha diversity variations across different saline–alkali habitats. **(a, c)** Venn diagrams of bacterial **(a)** and fungal **(c)** operational taxonomic units (OTUs). **(b, d)** Boxplots of α-diversity indices (Shannon, Simpson, Pielou) for bacterial **(b)** and fungal **(d)** communities.

Based on the Venn diagram, the bacterial OTU richness followed the order Shrub > Maize > SAF > Grass, with Shrub and Maize soils containing the higher numbers of unique OTUs (4,995 and 3,971 respectively; Figure 3a). The number of OTUs shared among all saline–alkali soil types was relatively low, accounting for 1.9% of all OTUs, and no OTUs were common across all saline–alkali soils. Analysis of the bacterial α-diversity showed that the Shannon, Simpson, and Pielou indices differed significantly among soil types (*P* < 0.05), with Shrub and Maize soils exhibiting higher values than Grass and SAF soils (Figure 3b).

The number of fungal ASVs showed the following trend: SAF > Shrubs > Maize > Grass (Figure 3c). The number of shared ASVs across all saline–alkali types was relatively low (3.3%), with the highest proportion of unique ASVs found in SAF (33.3%) and the lowest in grass (17.6%). The proportion of ASVs shared across all soil types was approximately 3.3%. No significant differences were found in the Shannon (*P* = 0.29), Simpson (*P* = 0.20), or Pielou (*P* = 0.22) indices across the soil types (Figure 3d). The Shannon index ranged from 2.13 to 5.07, the Simpson index ranged from 0.63 to 0.99, and the Pielou index ranged from 0.51 to 0.96.

### 3.4 Soil microbial co-occurrence networks

**Figure 4:**
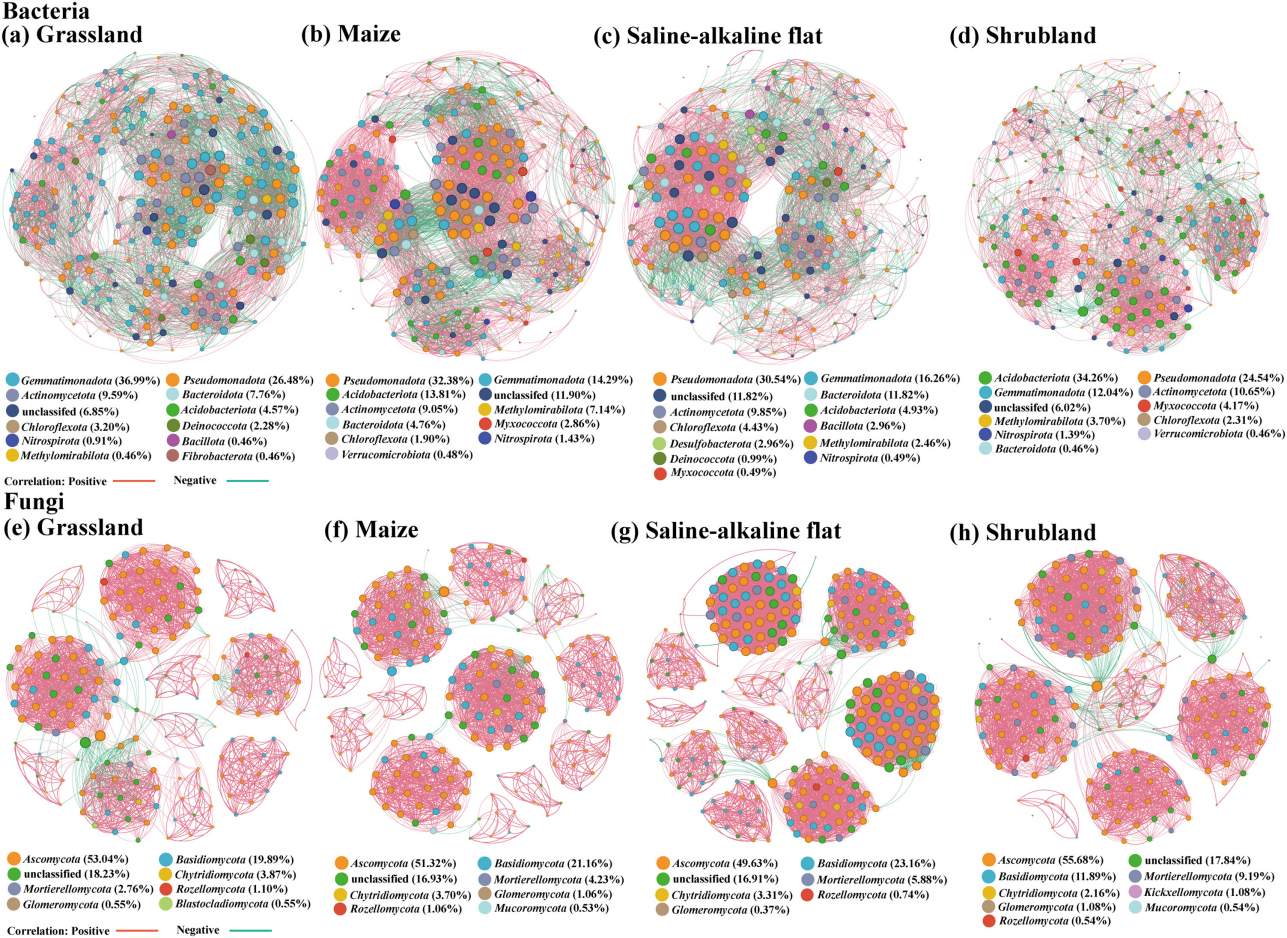
Co-occurrence networks of soil bacterial **(a–d)** and fungal **(e–h)** communities under saline–alkali stress. Colors of nodes represent different microbial phyla, and size of nodes correspond to the number of connections. Red lines indicate positive inter-node interactions (Spearman's *r* > 0.5, *P* < 0.05), while green lines represent negative ones between individual nodes (Spearman's *r* < −0.5, *P* < 0.05).

Bacterial co-occurrence network analysis revealed that the bacterial interaction networks tended to become simplified with enhanced competition under saline–alkali stress (Figures 4a–d). Maize soils exhibited the highest edge count (4,392), average degree (41.83), network density (0.20), and network complexity (0.64), reflecting the most frequent and closest microbial interactions (Table 2). Under saline–alkali stress, SAF soils showed a decrease in terms of edges, average degree, modularity, average path length and network complexity, suggestive of simplified bacterial interactions. Shrub soils had the highest modularity, average path length and positive correlation ratio, indicating stronger community stability and disturbance resistance. Grass soils had a positive correlation ratio of 59.06%, suggesting greater competition or antagonism among bacterial taxa.

**Table 2:** Network topological of bacterial community under saline–alkali stress.

Parameter (bacterial)	Grass		Maize		SAF		Shrub	
	Obs.	Perd.	Obs.	Perd.	Obs.	Perd.	Obs.	Perd.
Node	219	218	210	208	203	201	216	216
Edges	4,172	3,953	4,392	4,182	3,879	3,676	2,724	2,508
Modularity	0.56	0.52	0.53	0.50	0.50	0.43	0.66	0.61
Clustering coefficient	0.70	0.66	0.73	0.70	0.72	0.68	0.68	0.66
Average path length	2.84	1.88	2.84	1.85	2.82	1.88	3.27	2.04
Average degree	38.10	38.10	41.83	41.83	38.22	38.22	25.22	25.22
Network density	0.18	0.18	0.20	0.20	0.19	0.19	0.12	0.12
Positive correlation	59.06	59.06	76.96	76.96	71.59	71.59	80.43	80.43
Negative correlation	40.94	40.94	23.04	23.04	28.41	28.41	19.57	19.57
Complexity	0.61	0.61	0.64	0.64	0.46	0.46	0.44	0.44
**Parameter (fungi)**	**Grass**		**Maize**		**SAF**		**Shrub**	
	Obs.	Perd.	Obs.	Perd.	Obs.	Perd.	Obs.	Perd.
Node	181	181	189	189	272	272	185	182
Edges	2,040	1,859	2,070	1,881	4,581	4,309	2,785	2,600
Modularity	0.79	0.17	0.81	0.17	0.79	0.13	0.77	0.14
Clustering coefficient	0.95	0.17	0.97	0.15	0.98	0.17	0.96	0.19
Average path length	3.81	2.05	2.67	2.08	3.04	1.99	5.00	1.93
Average degree	22.54	22.54	21.91	21.91	33.68	33.68	30.11	30.11
Network density	0.13	0.13	0.12	0.12	0.12	0.12	0.16	0.16
Positive correlation	93.97	93.97	96.09	96.09	96.64	96.64	95.83	95.83
Negative correlation	6.03	6.03	3.91	3.91	3.36	3.36	4.17	4.17
Complexity	0.25	0.25	0.31	0.31	0.63	0.63	0.43	0.43

Obs. = observed value, Pred. = predicted value (mean of 999 random networks generated by the degree-preserving null model). All observed modularity, clustering coefficient, and average path length are significantly higher than null expectations (two-tailed *P* < 0.001). Average degree, network density, positive correlation, negative correlation and complexity are constant in the null models, thus predicted values equal observed values.

Fungal co-occurrence network formed a structurally complex and highly modular interaction system, showing topological characteristics distinct from those of the bacterial network (Figures 4e–h). Overall, the fungal networks showed high modularity (0.77–0.81) and clustering coefficient (0.95–0.98), forming highly compact modular structures. All fungal networks exhibited positive correlations (>93%), with synergistic coexistence as the dominant pattern. SAF soils host the largest fungal network (Node = 272, Edges = 4,581), with the highest interaction complexity (0.63), an average degree of 33.68, and a positive correlation ratio of 96.64%. Shrub fungal networks encompassed the highest number of phyla and showed the longest average path length, with network complexity of 0.43, indicative of a complex structure and high stability. Null model analysis revealed that the modularity, clustering coefficient, and average path length for both bacterial and fungal networks across all habitats were significantly higher than expected by chance (*P* < 0.001), confirming that the network structures were deterministic rather than random.

### 3.5 Functional prediction of soil microbial communities

**Figure 5:**
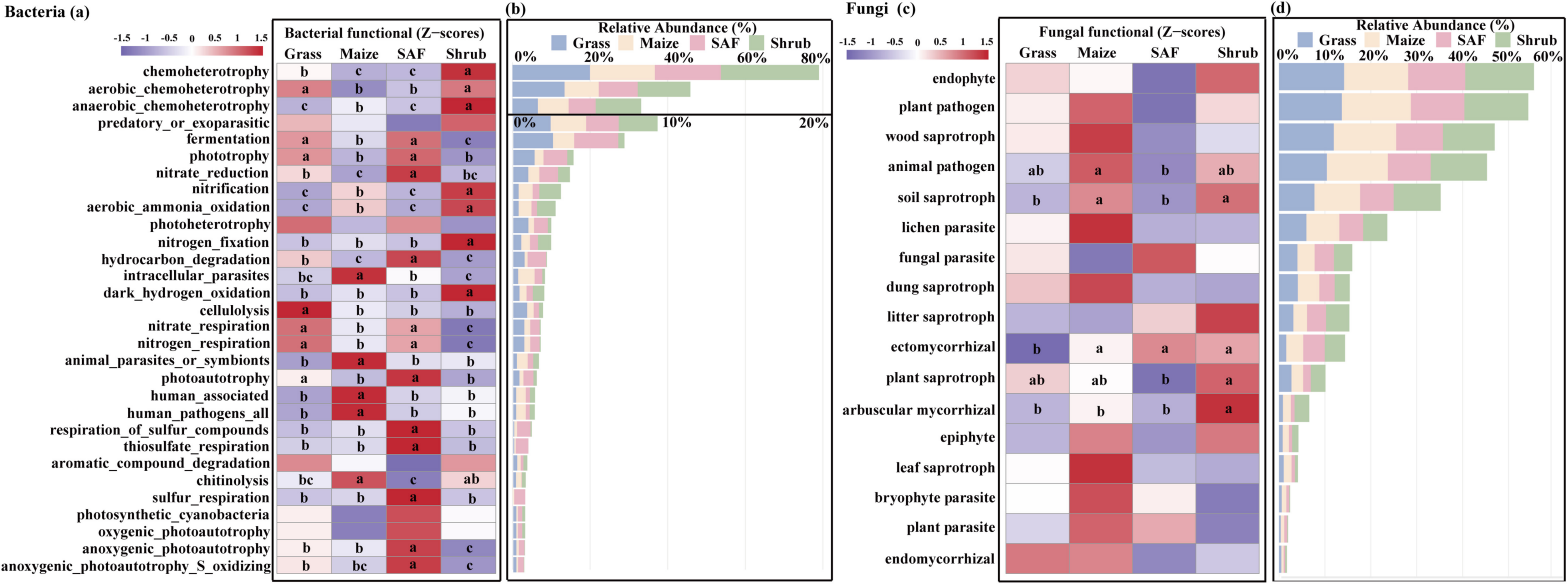
Functional prediction of soil bacterial **(a, b)** and fungal **(c, d)** communities under saline–alkali stress. **(a, c)** Heatmaps showing *Z*-scores of bacterial (FAPROTAX) and fungal (FUNGuild) functional groups, where red and blue indicate high and low abundance, respectively; different lowercase letters (a, b, c) indicate significant differences among habitats (*P* < 0.05). **(b, d)** Relative abundance of dominant bacterial and fungal functions, with different colors representing different functional groups.

Functional prediction based on the FAPROTAX database showed that soil bacteria in saline–alkali environments participated in 60 functional groups, accounting for 56% of all OTUs (Figures 5a, b). Under saline–alkali stress, bacterial community functions were dominated by heterotrophic metabolism, with chemoheterotrophy (19.75%), aerobic chemoheterotrophy (11.46%) and anaerobic chemoheterotrophy (8.28%) as core functions. The functional profiles differed significantly among soil types. Shrub soils exhibited the highest chemoheterotrophy and anaerobic chemoheterotrophy, while SAF soils were enriched in fermentation, phototrophy and nitrate reduction. Multiple nitrogen cycle-related functions were identified, including nitrate reduction, nitrification, aerobic ammonia oxidation and nitrogen fixation. Among those functions, nitrification, aerobic ammonia oxidation and nitrogen fixation were most abundant in Shrub soils.

FUNGuild-based predictions showed that fungi in saline–alkali environments were dominated by saprotroph (34.49%), followed by symbiotroph (21.41%) and pathotroph (19.94%; Figures 5c, d). Saprotroph was most abundant in Maize soils (37.44%), then Shrub soils (33.47%) and lowest in SAF soils (31.64%). Symbiotroph was enriched in Shrub and Maize soils but was lower in Grass and SAF soils. The dominant functional guilds included endophyte, plant pathogen, wood saprotroph, animal pathogen, soil saprotroph and lichen parasite, with showed distinct distribution patterns. Shrub soils were enriched in soil saprotroph, litter saprotroph and arbuscular mycorrhizal. Maize soils had higher plant pathogen, wood saprotroph, lichen parasite and arbuscular mycorrhizal. Grass soils were enriched for animal pathogen and dung saprotroph. SAF soils were characterized by fungal parasite.

### 3.6 Relationships between soil microbial communities and environmental factors

**Figure 6:**
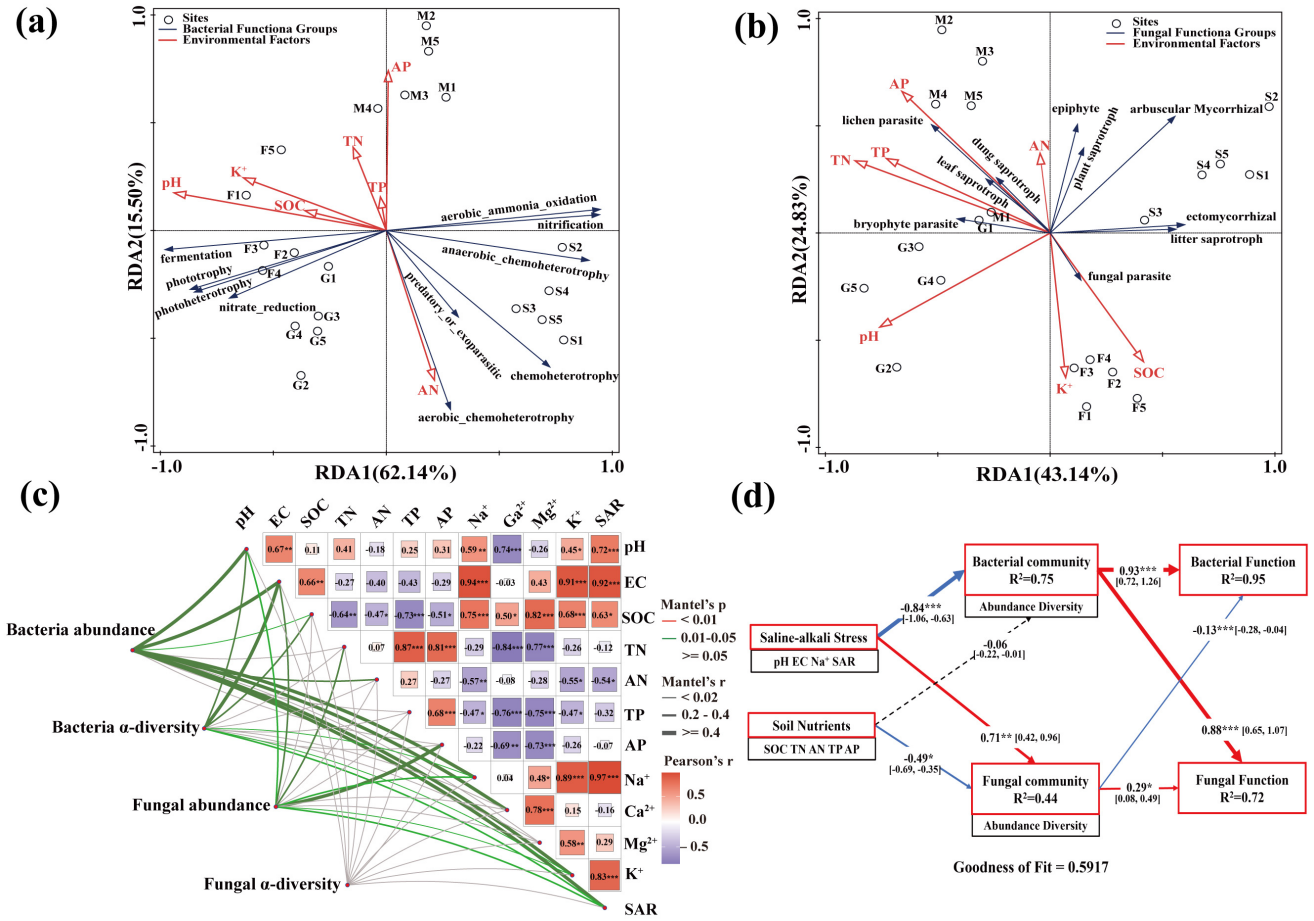
Mantel test and RDA of environmental factors with soil bacterial and fungal community composition. **(a, b)** RDA of bacterial **(a)** and fungal **(b)** functional groups constrained by soil physicochemical properties. **(c)** Mantel test and Pearson correlation heatmap and between soil physicochemical factors and bacterial/fungal abundance and α-diversity. **(d)** Partial least squares path model (PLS-PM) presenting the effects of saline–alkali stress and soil nutrients on soil microbial communities and functions. In **(a)** and **(b)**, environmental factor abbreviations are: SOC, soil organic carbon; TN, total nitrogen; AN, available nitrogen; TP, total phosphorus; AP, available phosphorus. Red and blue arrows in **(d)** indicate significant positive and negative effects (*P* < 0.05), respectively; gray arrows indicate non-significant effects. Goodness of fit = 0.5917. *R*^2^ values indicate the proportion of variance explained in endogenous variables.

After multicollinearity assessment (VIF < 10), seven factors (pH, SOC, TN, AN, TP, AP, and K^+^) were retained for RDA. The RDA model demonstrated a good fit, with the first two axes explaining 77.64% of the variation in bacterial functional groups (Figure 6a). Variance decomposition indicated pH, AP, and SOC as significant drivers (*P* < 0.05), explaining 55.4%, 15.5%, and 7.9% of variation, respectively, with pH as the dominant factor (pseudo-*F* = 22.4). Specifically, chemoheterotrophy, aerobic ammonia oxidation, anaerobic chemoheterotrophy, and nitrification functions correlated negatively with pH, whereas fermentation, phototrophy, photoheterotrophy, and nitrate reduction correlated positively with pH and SOC. RDA based on the seven VIF-screened environmental factors explained 67.97% of the variation in the fungal functional groups (Figure 6b). Among these factors, pH and AP were identified as significant drivers of the fungal functional group distribution (*P* < 0.05), with respective explanatory powers of 38.4 and 11.6%. Arbuscular mycorrhizal fungi, plant saprotrophs, and endophytes correlated negatively with pH, whereas lichen parasites, leaf saprotrophs, and dung saprotrophs correlated positively with AP, TP, and TN.

Mantel testing revealed that soil bacterial communities exhibited stronger responses to environmental factors than did fungal communities (Figure 6c). Bacterial abundances were significantly positively with pH, EC, SOC, SAR, AN, Na^+^, Ca^2+^, and K^+^ (*P* < 0.05), whereas α-diversity correlated positively with pH, EC, SOC, Na^+^, and K^+^ (*P* < 0.05). These findings indicate that salinization indicators (pH, EC, Na^+^, K^+^, and SAR) and SOC synergistically drove the bacterial community structure. Correlation analysis among environmental factors showed that key salinization indicators (pH, EC, Na^+^, K^+^, and SAR) were highly and positively correlated, revealing a synergistic relationship. Regarding fungal communities, fungal abundance correlated positively (*P* < 0.05) with pH, EC, TN, AP, Na^+^, K^+^, and SAR, whereas no significant associations were found between α-diversity and environmental factors. Our PLS-PM results showed that saline–alkali stress significantly inhibited bacterial communities while promoting the expansion of fungal communities and that soil nutrients negatively impacted fungal communities (Figure 6d). Both communities positively drove their corresponding functions (path coefficients of 0.93 of bacteria and 0.88 for fungi), with bacteria enhancing fungal function and fungi exerting negative feedback on bacterial function.

## 4 Discussion

### 4.1 Microbial community composition and diversity characteristics under saline–alkali stress

The results of previous studies conducted in representative salinized areas of this ecotone, such as Ningxia and the western Songnen Plain, demonstrated that soil salinization markedly altered the bacterial community diversity and composition, with the salinity gradient serving as the dominant factor shaping the microbial community structure (He et al., 2025; Tian et al., 2025). *Pseudomonadota* was the dominant phylum across all habitats, whereas *Gemmatimonadota* was enriched in Grass (34.90%) and *Bacteroidota* predominated in SAF (11.04%). This divergent pattern reflects the differential responses of microorganisms to salinity gradients. Structurally, this differentiation manifested as enrichment for halophilic and alkaliphilic functional groups. Under saline–alkali stress, different phyla (including *Bacillota, Patescibacteriota*, and *Verrucomicrobiota*) exhibited significant enrichment, adapting to these extreme environments through mechanisms such as osmoprotectant synthesis, ion homeostasis regulation, and maintained pH neutralization (Sousa et al., 2015; Zhang et al., 2019; Ezenwanne et al., 2025). Concurrently, salt-sensitive phyla such as *Acidobacteriota* exhibited low abundance, consistent with findings from Californian saline–alkali lands where long-term salt stress drives microbial community succession toward halophilic and alkaliphilic phyla (Malal et al., 2025).

In terms of fungal communities, *Ascomycota* enrichment in shrublands (58.77%) was related to its cellulose-degrading and saprotrophic nutritional strategies, driving soil carbon turnover through plant litter decomposition and mutualistic relationships with shrub roots that facilitate nutrient acquisition (Chaudhary et al., 2018; Zeng et al., 2020). *Basidiomycota* exhibited the highest abundance in SAF (17.08%) and correlated positively with soil Na^+^ and SAR (*P* < 0.05), representing the core salt-tolerant group enabling fungal community adaptation to saline–alkali stress. *Mortierellomycota*, a group of plant growth-promoting fungi capable of decomposing organic compounds and improving soil health (Ozimek and Hanaka, 2021), is primarily regulated by vegetation type and carbon source availability, rather than salinity alone. Its enrichment in Maize and Shrub soils reflects the selective recruitment of fungi by vegetation cover.

Similarly, bacterial diversity was relatively high in Shrub and Maize habitats with denser vegetation cover, confirming the protective effect of vegetation on bacterial communities. In a study conducted in the Songnen Plain soda saline–alkali lands, the presence of seven salt-tolerant plants significantly reduced soil salinity and alkalinity (compared with that in SAF soils), while concurrently enhancing nutrient levels and enzyme activities (Song et al., 2025). This improvement was mainly attributable to plant litter and root exudates (including organic acids, sugars, and amino acids), which provided sufficient carbon sources and energy for microorganisms, thereby promoting soil organic matter decomposition and mineralization (Liu X. et al., 2025). Fungal diversity, however, did not differ significantly among habitats, suggesting that fungal communities had greater environmental buffering capacity. Such functional redundancy likely underpins ecosystem stability under saline–alkali stress (Deng et al., 2019; Zhang et al., 2023).

### 4.2 Microbial co-occurrence networks and adaptive strategies under saline–alkali stress

The bacterial co-occurrence network in the SAF group exhibited significant reductions in connectivity, average degree, modularity, and network complexity, accompanied by a relatively high proportion of negative correlations. These data indicate that the saline–alkali environment simplified the structure of bacterial interaction networks and intensified interspecific resource competition. The simple cellular structure of prokaryotic bacteria makes them highly sensitive to salinity stress. Elevated external osmotic pressure alters the permeability of bacterial cell membranes and causes the dysfunction of intracellular enzymes (Ali et al., 2023), whereas soil nutrient deficiency restricts their energy supply (Liu et al., 2022). To adapt to stressful environments, bacteria reallocate energy from growth metabolism to osmoregulatory processes, resulting in decreased overall metabolic activity and viability, ultimately reducing species diversity and decreasing bacterial network complexity (Wang et al., 2025).

In contrast, fungal networks in saline–alkali soils exhibited high modularity, high clustering coefficients, and predominantly positive correlations, most pronounced in SAF habitats. These findings indicate that fungi maintain functional stability under extreme stress through a “cooperative-coexistence” strategy characterized by highly synergistic and modularly integrated interspecific interactions. The complexity of SAF fungal networks stems from multilevel adaptive responses in genetics, metabolism, and hyphal structures. Halotolerant fungi can defend against ROS damage induced by saline–alkali stress by regulating cellular osmotic pressure, stabilizing cell membranes, and synthesizing antioxidant enzymes such as catalase (Gostincar and Gunde-Cimerman, 2018; Yovchevska et al., 2025). However, stress-adapted metabolism can reduce growth rates, and excessive ROS under severe stress can cause damage or even fungal and plant death (Hasanuzzaman et al., 2021). Additionally, fungal hyphae can penetrate soil pores to acquire nutrients, and their networks can enhance soil aggregation and promote nutrient transformation through extracellular enzyme secretion (Ramette and Tiedje, 2007; Li et al., 2019).

### 4.3 Soil microbial functional differentiation in saline–alkali habitats and its environmental drivers

Land-use change in the saline–alkali habitats of the agro-pastoral ecotone drove significant differentiation in the soil microbial functional profiles. Shrubs, characterized by balanced nutrients and lower pH values, avoided the inhibition of nitrogenase activity by excessive alkalinity (Oduor et al., 2025), thereby supporting the highest abundance of nitrification, aerobic ammonia oxidation, and nitrogen fixation functional groups. *Pseudomonadota* and *Actinomycetota* were identified as key nitrogen-fixing taxa. Rhizobia (belonging to *Pseudomonadota*) can establish symbiotic nitrogen-fixation systems with host plants, thereby enhancing microbial activity (Kim et al., 2021). Maize fields showed significantly increased nitrification, aerobic ammonia oxidation, intracellular parasitism, and human-associated functional groups than did the other habitats. Agricultural activities, such as fertilization and tillage, improved nitrogen transformation efficiency and promoted enrichment for human–animal symbiotic bacteria, forming a specific functional pattern distinct from that of their natural habitats. SAF, with high salinity and alkalinity, can suppress most heterotrophic bacteria, whereas phototrophs and fermenters can adapt to extreme environments through photosynthetic carbon fixation and anaerobic fermentation. The high-salt and high-alkali environment of SAF inhibits the growth of most heterotrophs, whereas phototrophic and fermentative bacteria adapt to extreme environments by obtaining energy through photosynthetic carbon fixation and anaerobic fermentation (Zhang et al., 2026). Metagenomic sequencing confirmed that these metabolic adaptations increased the abundance of microbial genes related to CO_2_ fixation, consequently strengthening the soil carbon-sequestration capacity (Wang and Kuzyakov, 2024; Long et al., 2025). The positive correlation between phototrophic and fermentative functional taxa and SOC further demonstrated that these groups can alleviate carbon limitations under salt stress by driving organic carbon accumulation. PLS-PM analysis revealed that land-use change promoted functional differentiation by optimizing the bacterial community structure (path coefficient = 0.93), explaining the ecological effects of enhanced bacterial diversity from SAF to Maize and Shrub.

The dominance of saprotrophs in fungal communities is attributable to their strong capability for decomposing organic matter. Saprotrophs showed the highest relative abundance in Maize, with wood, dung, and leaf saprotrophs positively correlating with AP, TP, and TN, indicating that agricultural organic inputs (e.g., straw return) provided resource bases for saprotrophic fungal enrichment (especially for *Ascomycota*). Pathotrophic fungi were also more highly abundant in Maize, reflecting enhanced fungus–plant interactions and indicating potential disease risks (Anthony et al., 2017). Notably, beneficial fungi, such as *Mortierella* (relative abundance: 13.96%) and *Aspergillus* were enriched in this habitat, potentially enhancing pathogen resistance through antagonistic effects (Moussaid et al., 2025; Xu et al., 2025; Liang et al., 2025). Vegetation improvement in saline–alkaline soils can create conditions supporting the colonization of beneficial endophytic fungi, thereby enhancing host stress tolerance through phytohormone and soil nutrient regulation (Jin et al., 2024). However, our RDA showed that saline–alkali stress inhibited the abundance of arbuscular mycorrhizal fungi and endophytes. The abundance of endophytic fungi in Shrub, Grass, and Maize habitats was higher than that in the SAF habitat, suggesting that land-use changes partially mitigated the stress effects.

PLS-PM analysis showed that bacterial communities significantly enhanced fungal function, whereas fungal communities impeded bacterial function, indicating an asymmetric functional regulatory relationship between both groups. Soil bacteria and fungi can collectively shape community assembly and ecological functions through niche differentiation, functional complementarity, and interspecific antagonism (Pawlowska, 2024). Bacteria predominantly utilize labile carbon sources, whereas fungi degrade recalcitrant organic matter (Jílková et al., 2019; Wang and Kuzyakov, 2024; Wang et al., 2025). Under saline–alkali stress, fungi demonstrate a greater stability and competitive advantage, providing substrates for bacteria by decomposing complex organic matter and regulating bacterial community assembly (Li et al., 2024; Gao et al., 2022; Jiao et al., 2022). In parallel, fungi secrete antimicrobial substances that can suppress the excessive expansion of bacterial communities, preventing the overexploitation of resources under stressful conditions. This synergistic bacterial–fungal relationship facilitates essential nutrient acquisition and alleviates salt stress. Some fungal functions identified in this study remain unclassified, indicating their potential for further exploration.

### 4.4 Research limitations

We performed high-throughput amplicon sequencing to characterize soil microbial community structures. Although this approach comprehensively captured species composition and diversity, it could not directly determine functional gene expression or metabolic activity. Our functional predictions were based on the FAPROTAX and FUNGuild Databases, the reliability of which was constrained by the completeness of the reference databases and lacked validation by metagenomic or culture-dependent experiments. Furthermore, sampling at a single time point (September 2024) precluded capturing the seasonal dynamics of saline–alkali stress.

## 5 Conclusion

The results of this study revealed the adaptive succession patterns and community-interaction mechanisms of soil microorganisms under saline–alkali stress in an agro-pastoral ecotone. The bacterial communities were sensitive to saline–alkali stress, exhibiting simplified community structures and intensified network competition, and achieved adaptive succession through the enrichment of salt-tolerant taxa such as *Bacillota, Patescibacteriota* and *Verrucomicrobiota*. In contrast, fungi formed highly modular and synergistic interaction networks that helped maintain their functional stability through cooperative coexistence, with *Basidiomycota* identified as an enriched halophilic fungal phylum. Saline–alkali stress significantly inhibited most bacterial metabolic functions, including chemoheterotrophy and nitrification, although it promoted fermentation, phototrophy, and nitrate reduction. Saline–alkali stress suppressed the growth of bacterial communities while promoting that of fungal communities, with both microbial groups positively contributing to their respective functional profiles. The transition from SAF to Shrub and Maize significantly alleviated saline–alkali stress and optimized microbial community structure and function.

Future research should focus on specific functional groups, particularly phosphate-solubilizing and nitrogen-fixing taxa, to clarify their regulatory mechanisms and provide targeted theoretical support for the ecological restoration and sustainable management of saline–alkali ecosystems.

## Data Availability

The data presented in the study are deposited in the NCBI repository, accession number PRJNA1440631.
